# A New Device to Mimic Intermittent Hypoxia in Mice

**DOI:** 10.1371/journal.pone.0059973

**Published:** 2013-04-02

**Authors:** Kamil J. Chodzyński, Stephanie Conotte, Luc Vanhamme, Pierre Van Antwerpen, Myriam Kerkhofs, J. L. Legros, Michel Vanhaeverbeek, Alain Van Meerhaeghe, Gregory Coussement, Karim Zouaoui Boudjeltia, Alexandre Legrand

**Affiliations:** 1 Fluid-Machines Department, University of Mons, Mons, Belgium; 2 Physiology and Pharmacology Department, University of Mons, Mons, Belgium; 3 Laboratory of molecular parasitology, IBMM, Free University of Brussels, Brussels, Belgium; 4 Laboratory of therapeutic chemistry, Faculty of Pharmacy, Free University of Brussels, Brussels, Belgium; 5 EPHEC, ISAT, Schaerbeek, Brussels, Belgium; 6 Sleep Laboratory, CHU de Charleroi, Montigny-le-Tilleul, Belgium; 7 Experimental Medicine Laboratory (ULB 222 Unit), CHU de Charleroi, Montigny-le-Tilleul, Belgium; National Institutes of Health, United States of America

## Abstract

Intermittent hypoxia (hypoxia-reoxygenation) is often associated with cardiovascular morbidity and mortality. We describe a new device which can be used to submit cohorts of mice to controlled and standardised hypoxia-normoxia cycles at an individual level. Mice were placed in individual compartments to which similar gas flow parameters were provided using an open loop strategy. Evaluations made using computational fluid dynamics were confirmed by studying changes in haemoglobin oxygen saturation *in vivo*. We also modified the parameters of the system and demonstrated its ability to generate different severities of cyclic hypoxemia very precisely, even with very high frequency cycles of hypoxia-reoxygenation. The importance of the parameters on reoxygenation was shown. This device will allow investigators to assess the effects of hypoxia–reoxygenation on different pathological conditions, such as obstructive sleep apnoea or chronic obstructive pulmonary disease.

## Introduction

Hypoxia-reoxygenation (also referred to as intermittent hypoxia [IH]) is a process whereby phases of low tissue oxygen pressure (hypoxia) alternate with phases of normal tissue oxygen pressure (normoxia). This phenomenon is associated with pathological conditions, such as myocardial infarction, stroke, cardiac arrest or septic shock [1], [2]. Respiratory disorders, such as obstructive sleep apnoea (OSA) or central sleep apnoea, are characterised by repeated bouts of hypoxia [3]. In such diseases, the partial oxygen pressure fluctuates during sleep and cyclic tissue hypoxia develops, sometimes occurring as often as 60 times per hour. The deleterious consequences of this phenomenon appear to originate from both the hypoxic phase and the reoxygenation process. Prolonged falls in oxygen concentration lead to pulmonary vascular remodelling, elevated pulmonary artery pressures and right heart hypertrophy. But, when coupled with abrupt reoxygenation, hypoxia is associated with oxidative stress and an inflammatory response, which have multiple cardiovascular, neurocognitive and metabolic sequelae [4], [5]. Nevertheless, the molecular mechanisms supporting such pathophysiological effects remain incompletely understood *in vivo*.

This incomplete knowledge is partly related to technical problems. Oxygen depletion can be achieved by supplementation of nitrogen in the inspired air. Multiple protocols have been reported with major differences regarding the depth and duration of hypoxia (from seconds [6] to an hour [7]), the number and length of hypoxia-reoxygenation cycles (from tens [8] to tens of thousands [6]), and the total period of exposure to these cycles. In addition, the changes in oxygen tension in these devices were slow and the times spent at the desired low or high oxygen pressure were limited and varied. Furthermore, although gas flow can be controlled in customised cages, homogeneity of the gas mixture is difficult to achieve, because of the laws of fluid mechanics leading to non-laminar (disturbed) flow inside the cage. It cannot, therefore, be guaranteed that different mice, despite being in the same cage, will be submitted to the same air-conditions. In rat studies, attempts to eliminate these drawbacks have been conducted by placing a hood around the animal’s head or using cylindrical Plexiglass chambers of smaller volume allowing a higher cycle frequency without reduction of the oxygen nadir [6], [8]. Animal models now recourse more frequently to mice. We describe a new device in which mice can be kept in individual compartments and submitted to similar standardised and controlled air-flow conditions, mimicking those found in clinical conditions.

## Materials and Methods

### Gas mixture and Mouse Chamber Design and Set Up

**Figure 1:**
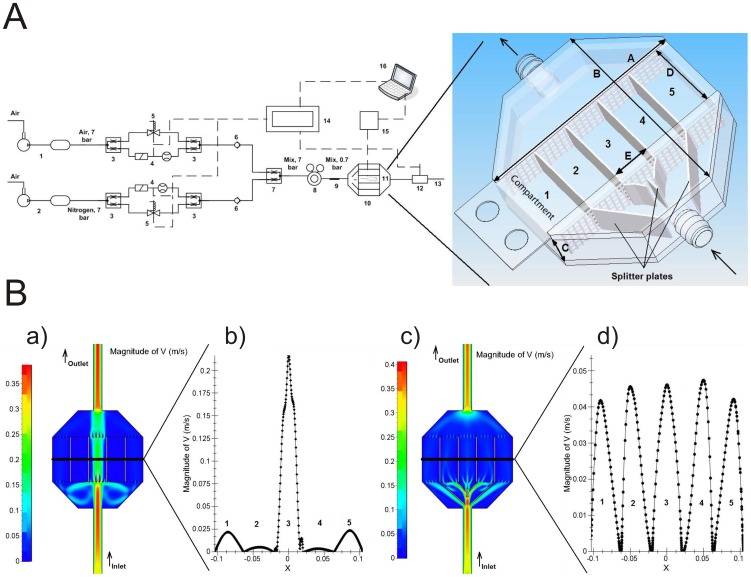
A device providing reliable and homogenous gas concentrations in individual mouse rooms. A: Technical scheme of the circuitry 1) air compressor and tank, 2) nitrogen compressor and tank, 3) T-connectors, 4) flowmeter with an analogue valve, 5) solenoid valve, 6) check valve, 7) Y-connectors, 8) pressure regulator, 9) static mixer, 10) mouse cage, 11) mouse, 12) oxygen sensor, 13) outlet of gas mixes, 14) controller with touch panel, 15) device to measure arterial blood oxygen saturation, 16) notebook to control, visualize and save the data. Solid line: pipes, dashed line: electrical wires. B: Insertion of splitters in the airflow allows gas concentrations to be homogenised within the mouse chambers: CFD simulation of the gas flow in the mouse box without (a) and with (c) splitters; velocity profiles inside each box without (b) and with (d) splitters (section cut).

In contrast to previous approaches to the problem, our model is based on laminar flows and infusion of carefully determined gas mixtures. The circuitry of the device is shown in Figure 1A. The movement of an injection-fluid front through the chamber allows a rapid change in the inhaled-gas composition. To achieve this, gas flow conditions are formed by two parallel flow lines: One line supplies air containing 21% oxygen and 79% nitrogen and is fed by a compressor tank (1, Contimac CM 403/10/100 W, capacitance 100 L); the second line supplies the system with nitrogen (99.5%) provided by a nitrogen compressor tank (2, Nitrocraft NC LC.MS 013C, capacitance 500 L). Both tanks are pressured at an effective pressure level of 7 bars. The two lines are then split into two half-flows using a T-connection (3). For both lines, the first half-flow is directed to a flowmeter with an adjustable valve (4, Brukert, 8711 MFC 10 L/min). The second half-flow is directed through a solenoid DC valve (5, Brukert, 6027 A 4.0 FKM VA). The two half-flows are then joined again using a T-connection (3). To avoid back flow, check valves (6) are placed downstream of the T connections. The two lines are then joined together into a single flow mixture through a Y-connection (7). The gas flow is then driven through a gas regulator (8, Masterflex, Single-Stage Regulator, 98202–03) to reduce the pressure from 7 bars to 0.7 bars. A static mixer (9) is then inserted in order to ensure the homogeneity of the mix. The resulting gas mixture (containing a specified oxygen level) is then driven through the plastic mouse cage (10) divided into 5 independent compartments each designed to fit one mouse (11). The oxygen level is measured by the oxygen sensor (12, Sensortechnic, the oxygen sensor XYA5M with the circuit board ZBXYAF) placed downstream of the cage. Finally the gas mixes are released. The whole system is controlled by a real-time device (14, E.D.&A., High Professional Controller model V5–19). Mouse behaviour is monitored by a specific device (see below). A notebook connected to the system enables the data to be recorded, saved and visualized for further processing.

Computational fluid dynamics (CDF) calculations were performed using the Fine Hexa 2.10 Numeca software. Simulations were performed using a 2D model, thus neglecting the upper (roof) and lower (ground) wall effects. They were performed using a 2D air laminar flow of 5 l/min at 20°C and atmospheric pressure. These simulations were performed on empty compartments, thus neglecting the possible influence of animal movement on flow conditions. Given the parameters of the system (low flow rate, length and diameter of the pipes, size of the box, used devices) we chose an open loop strategy to assess its efficacy. This control system was divided into two parts: the first part responsible for controlling the oxygen levels between 21 and 13%, and the second part to control them between 12-0 %. The division was forced by a real-time controller able to open the DC valve in a minimum time of 20 ms for all possible sets of instructions. As this time is too long to adequately control the reference signal above the 12% oxygen concentration, a small overshoot on the minimum level of the oxygen was provided by the system. The valves directing the two parts of the system are controlled using the equations below. Taking into account the flow rate conservation law, the flow rate of mixing lines is the sum of the two incoming flow lines:
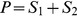
(1)


From mass conservation and concerning a perfect mixing of the two incoming line, for desired flow rate of the product mixing line (P) and desired oxygen level C_d_, we can write:
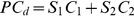
(2)


Then from equation (1) the flow rate S_1_ (l/min) for the normal air line is calculated as:
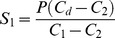
(3)


Above equations are limited to the oxygen level:
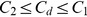
(4)


As well as flow rate
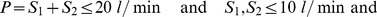
(5)Where:

P is the flow rate of the mixing line (product)

S_1_ is the flow rate of the normal air line

S_2_ is the flow rate of the nitrogen line

C_d_ is the minimum oxygen concentration of the product line

C_1_ is the oxygen concentration in the normal air line

C_2_ is the oxygen concentration in the nitrogen line

**Figure 2:**
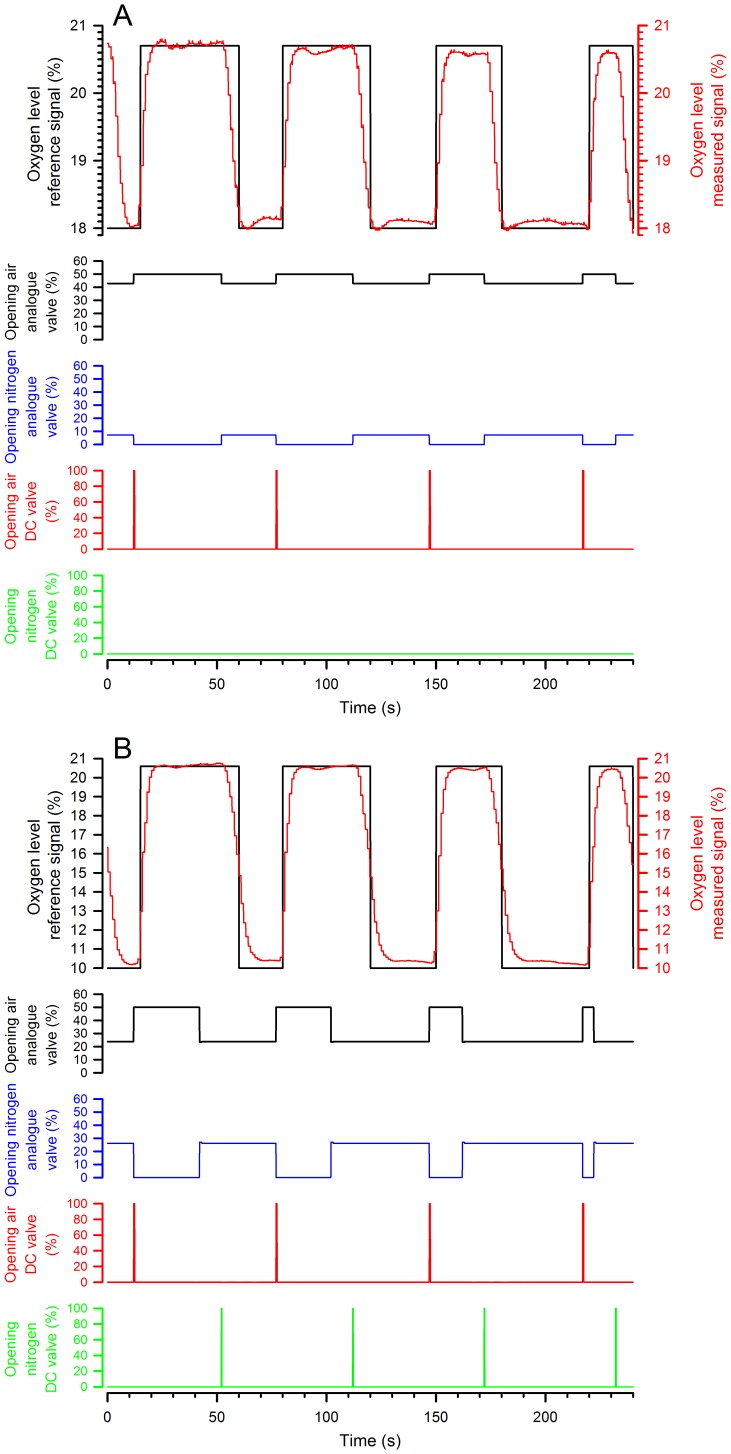
Control of the DC and analogue valves as a function of the required FIO_2._ The traces illustrate the strategy used when the required FIO_2_ was between 21%-13% (here 18%, A) or 12-0% (here 10%, B). The first graph corresponds to traces of expected and measured gas concentrations, and the others to the operating signals controlling the valves.

The user has to set the desired flow rate of the product line (P) and a minimum oxygen concentration of the product (C_d_). The real-time controller then measures the oxygen concentration in line 1 (C_1_) and line 2 (C_2_) as an average of measurements of 1000 values. The controller then monitors the reference signal of the desired oxygen concentration (C_d_) and calculates the line 1 (S_1_) and the line 2 (S_2_) flow rates based on equations (1) and (2). However, as the analogue valves do not respond fast enough to the requested signal (especially at minimum and maximum values), we decided to add DC valves controlling normal air for the first strategy (when the required FIO_2_ was between 21%-13%, Figure 2 A) and both normal air and nitrogen for the second one (when the required FIO_2_ was between 12%-0%, Figure 2 B). These valves decrease the time needed to reach critical points of the control signal because they open in advance and for long enough to obtain a minimum nitrogen level and a maximum oxygen level.

The currently firmware that is installed in the system is able to realize the number of cycles between 1 and 9999 with the FIO2 between 21% and 0.5% and the different time periods of 15 s hypoxia - 45 s normoxia, 20 s hypoxia - 40 s normoxia, 30 s hypoxia - 30 s normoxia, 40 s hypoxia - 20 s normoxia. Moreover, it is also possible to do test with different FIO2 (21%, 18%, 15%, 12%, 9%, 6%) during 3 minutes.

New experiments were performed in order to answer that question. Thus, to evaluate the system, partial oxygen pressure was measured downstream of the cage (using the oxygen sensor XYA5M with the circuit board ZBXYAF, Sensortechnic™, Munich, Germany). The actual inspiratory fraction of oxygen (FIO_2_) and the time needed to reach a stable composition (±1%) were evaluated for the different FIO_2_ values required (21%, 18%, 15%, 12%, 9% and 6%). The actual FIO_2_ values were very similar to the desired values (Table 1a) are now indicated for every mixture tested (Table 1b). *In vivo* analyses: material and design.

**Table 1:** Evaluation of the device’s ability to provide the required FIO_2_.

A.						
Desired FIO_2_	21%	18%	15%	12%	9%	6%
Actual FIO_2_ (%)	20.6 ± 0.1	17.5 ± 0.3	14.6 ± 0.2	11.8 ± 0.2	8.8 ± 0.2	5.9 ± 0.3
**B.**						
FIO_2_ to reach	18 ± 1 %	15 ± 1 %	12 ± 1 %	9 ± 1 %	6 ± 1 %	
Time to reach the FIO_2_	4.08 ± 0.9 s*	10.6 ± 0 s	9.12 ± 1 s	9.78 ± 0.9 s	14.84 ± 4.2 s*	

A) Evaluation of actual inspiratory fraction of oxygen (FIO_2_) for the different FIO_2_ values tested. Values are expressed as mean ± SD.

B) Time necessary to reach a stable composition (± 1%) for the different FIO_2_ values tested.

ANOVA on ranks was used as the statistical test. Values are expressed as mean ± SD. p values were < 0.05;

* Indicates significant difference (p<0.001) between the time to reach 18 ± 1 % and 6 ± 1%.

A biological validation of the model was obtained by transcutaneous measurements of the oxygen saturation of arterial haemoglobin during exposure. All procedures met the standards of the national Belgian requirements regarding animal care and were carried out in accordance with the Animal Ethics and Welfare Committee of the University of Mons. NMRI mice, weighing 30–54 g, were obtained from an internal animal breeding facility. The mice were housed using a 12:12 h (light:dark) photoperiod and were given unrestricted access to food and water except during the experimental periods. During the experiments, mice were not fed or watered within the device. Pulse oxygen saturation levels were recorded using an oximeter placed around the animal’s neck. This device integrates a spectrophotometer and a photoplethysmograph (Collar Clip^TM^, MouseOx® with Software 6.20; Star™ Life Sciences Corp. Oakmont, PEN). All efforts were made to minimise stress and animals were sedated with an intraperitoneal injection of ketamine (Ketalar® Pfizer, 50 mg/kg of body weight) and xylazine (Sigma-Aldrich, 10 mg/kg).

In a group of 7 animals, we first evaluated the degree of desaturation when a stable low oxygen pressure was acutely applied to the chamber for 3 minutes. The animals were then exposed to cyclic hypoxia with the different FIO_2_ values mentioned above. The duration of the cycle was fixed at 60 seconds. A synchronization signal was first emitted. The hypoxic phase was then progressively lengthened from 15 to 20, 30 and 40 seconds during four consecutive cycles. The chronology (latency and time to nadir) and the amplitude of desaturation and resaturation were, therefore, studied as a function of the duration of the hypoxic phase. For the 12% FIO_2_, the measurements were repeated in positions 1, 3 and 5 of the box. Three cycles of each hypoxic-phase duration were studied. Finally, as a stable level of desaturation/resaturation was not reached after 3 cycles in some conditions, these measurements were repeated during chronic cyclic hypoxia (10-minute duration recordings). All measures were made in triplicate.

### Data Analysis and Statistics

The saturation data were submitted to signal extraction to eliminate erratic data (software error code > 3). Data were averaged and are expressed as mean ± SD. Statistical analysis was performed using an ANOVA on rank or a one-way ANOVA followed by a Newman-Keuls test (Sigma Plot 12.3 Software) when appropriate. A p value < 0.05 was considered as significant.

## Results

The circuitry of the device is detailed in the material and methods section and schematised in Figure 1A. It ultimately leads into the customised cage divided into 5 compartments each allowing a single mouse. A series of questions was asked to assess the validity of the device design and of the CFD simulations.

Firstly, is it possible to generate similar flows in the five individual compartments? To equally divide the main flow otherwise directed at the central room (Figure 1Ba and 1Bb), five splitter plates were introduced into the device next to the gas inlet. We used an empirical procedure to adjust the plate angles and dimensions and performed CFD simulations for each new splitter plate geometry until division of the flow into 5 smaller components was optimal (Figure 1Bc). Velocity profile analysis (Figure 1Bd) indicated that this ultimately led to the same conditions in all compartments with a difference of no more than ± 10% in each room compared to the mean velocity.

Secondly, does the oximetry reliably mimic the required oxygen signal? To answer this question, oxygen levels were recorded using the oxygen sensor located at the exit of the box. For this purpose, we programmed the oxygen input to be delivered as a square wave signal (Figure 2) for 4 cycles of 60 seconds each. Different cycle matrices were designed and performed in three independent tests. The results showed that the oxygen levels closely followed the modelled concentrations in the configurations used to generate high and low oxygen concentrations (Figure 2 A and B). The four operating curves of Figure 2 A and B show the operating signals controlling the valves opening that were used to generate the records.

Difficulties in changing oxygen levels rapidly are intrinsic to fluid dynamics and related to changes in pneumatic line geometry, viscosity and compressibility of the medium. When the mouse box oxygen concentration was compared to that in the pneumatic low pass-filter in the pneumatic line, the average difference compared to the reference signal was about 5.7 ± 0.6%. This is likely the results of cuts at higher frequencies (an intrinsic property of this kind of filter), which have a big influence on the control loop.

Thirdly, can we rely on the FIO_2_ generated (the signal generated by the device) to determine the physiologically important parameter, i.e., the level of haemoglobin oxygen saturation. To address this question, pulse oximetry was measured after stabilisation of the saturation in 7 animals. We measured haemoglobin oxygen saturation after 3 minutes of exposure to an FIO_2_ of 21%, 18%, 15%, 12%, 9% and 6%.

**Figure 3:**
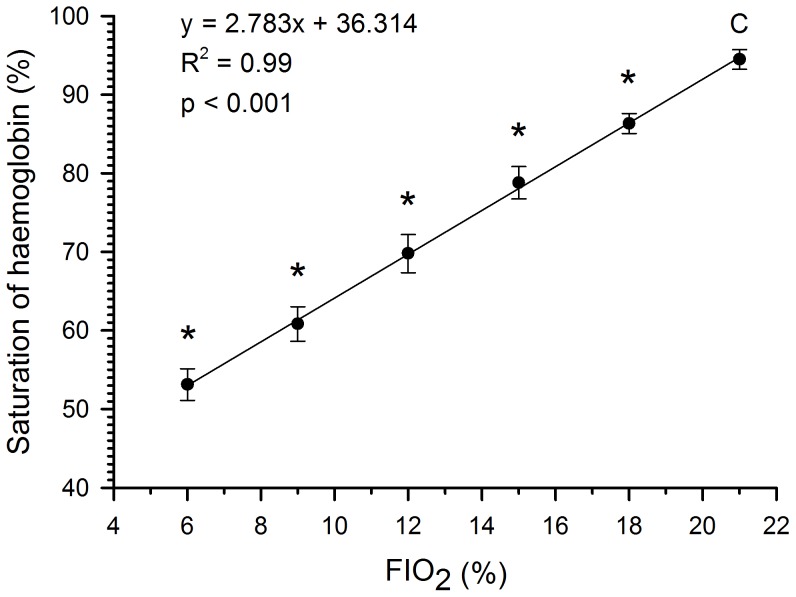
Oxygen saturation of haemoglobin as a function of FIO_2_. Values of haemoglobin oxygen saturation are measured after 3 minutes of exposure to a FIO2 and expressed as means ± SD for the different FIO_2_ values. Comparisons were made using a one-way ANOVA followed by Newman-Keuls test. *p<0.05.

**Figure 4:**
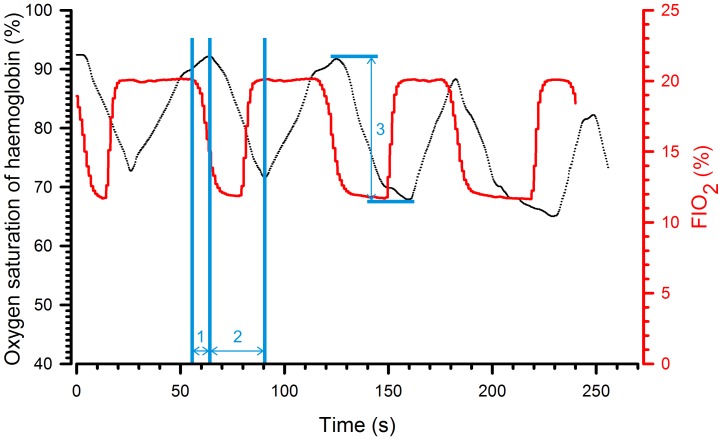
Evolution of haemoglobin oxygen saturation in response to a cyclic change in FIO_2_. Traces from a representative animal. Saturation was assessed during four consecutive cycles of 60 s during which the hypoxic phase was progressively lengthened (15, 20, 30 and 40 s). The red line corresponds to FIO_2_ in the inspired air, the black line to the transcutaneous measurement of haemoglobin oxygen saturation of the animal. Latency (1); time to peak (2) and amplitude of change in saturation (3).

Figure 3 indicates that a significant increase in haemoglobin oxygen saturation was observed for all FIO_2_ increments from 6% to 21% (p<0.001) and that there was indeed a linear correlation when haemoglobin saturation was plotted as a function of FIO_2_. Figure 4 superimposes the traces of FIO_2_ and haemoglobin oxygen saturation during cyclic hypoxia in a representative animal. The saturation did not reach equilibrium during the hypoxic phase (FIO_2_ 12%). In addition, the longer the hypoxic phase, the lower was the nadir. However, the nadir alone was not sufficient to describe the evolution of saturation. Before continuing, we, therefore, defined three parameters: the latency, which is the time between change in FIO_2_ and the beginning of the change in saturation; the time to peak, which is the time elapsed between the first and maximal change in saturation; and the amplitude of change, which is the difference in saturation between the first and the maximal change in saturation.

**Figure 5:**
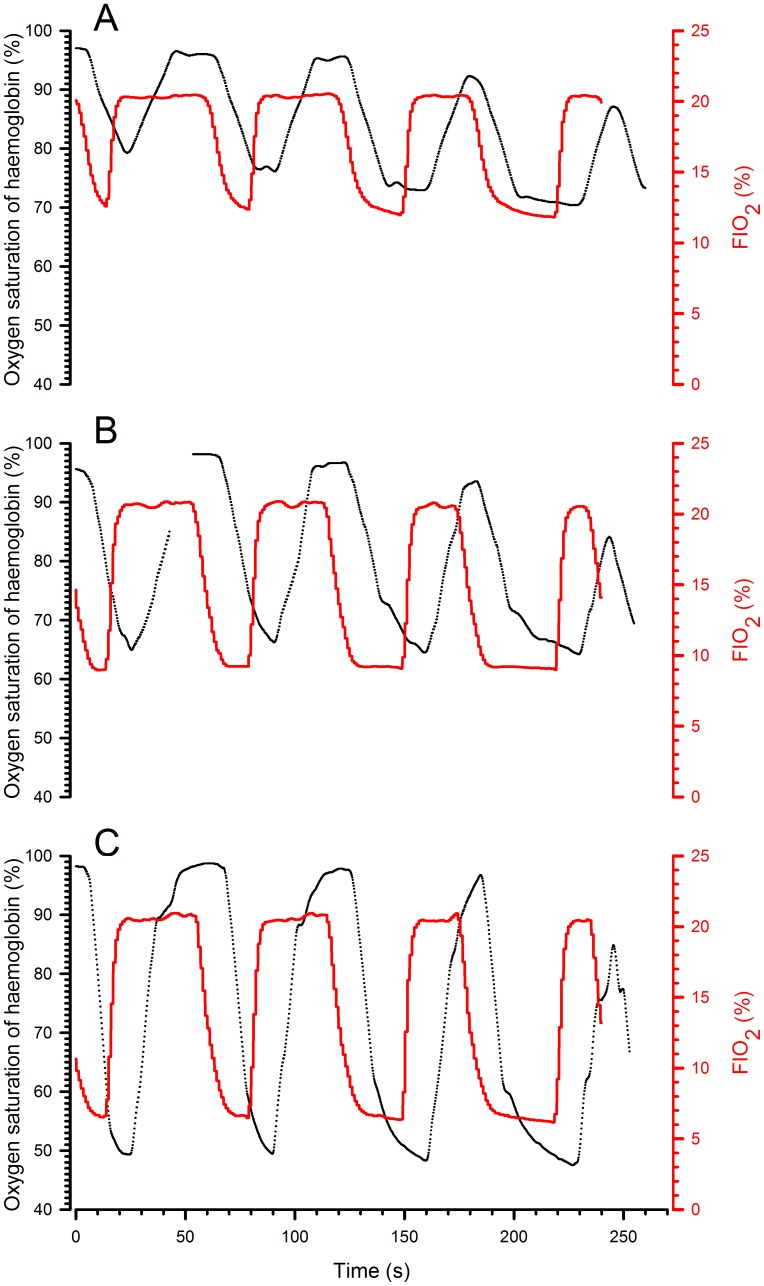
Representative records of haemoglobin oxygen saturation during hypoxia–reoxygenation with different durations of the hypoxic phase. During the four consecutive 60 s cycles, the hypoxic phase was progressively lengthened (15, 20, 30 and 40 s). The black line corresponds to the oxygen saturation of haemoglobin, the red line to FIO_2_ in the inspired air. The three graphs represent the cycles with different FIO_2_ values during the hypoxic phase (A: 12%; B: 9%; C: 6%).

Fourthly, does the system allow a cyclic change in oxygen saturation of arterial haemoglobin? In other words, can we design cycles allowing desaturation/resaturation (two important parameters in a hypoxia-reoxygenation model) according to different FIO_2_ values? To evaluate the effect of the hypoxic-phase duration on desaturation and resaturation, FIO_2_ values of 6%, 9% and 12% were applied according to the following cycles: (1) 15 seconds hypoxia – 45 seconds normoxia, (2) 20 seconds hypoxia – 40 seconds normoxia, (3) 30 seconds hypoxia – 30 seconds normoxia, (4) 40 seconds hypoxia – 20 seconds normoxia. A typical result is shown in Figure 5 and quantifications are reported in Table S1.

When the results obtained with the different FIO_2_ values and the different durations of the hypoxic phases were compared (Table S1), there were no significant differences in latency. With increasing hypoxic-phase duration, there was a significant difference in the time to peak desaturation and the time to peak resaturation. In addition, the desaturation and resaturation amplitudes were larger as the FIO_2_ decreased. As stated in the results section, a decrease in the amplitude of resaturation was observed when the normoxic phase was reduced to 20 s, independently of the applied FIO_2_.This, indicate that this period of time was not sufficient to achieve a complete resaturation of haemoglobin.

**Figure 6:**
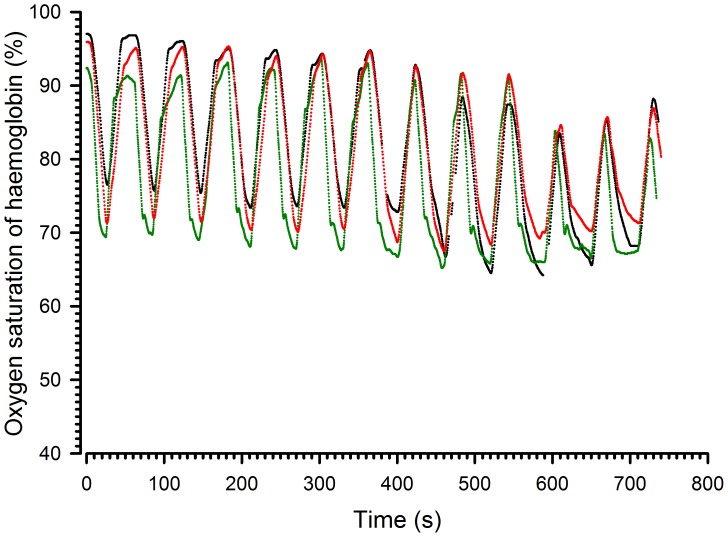
Representative records of haemoglobin oxygen saturation at different positions in the chamber. The black line corresponds to box 1, the green line to box 3 and the red line to box 5. FIO_2_: 12% alternating with 21% every 30 s.

Fifthly, does the homogeneity of the airflow translate into similar haemoglobin saturation levels in the different compartments? To address this question, the mice were placed in the peripheral and central compartments and measurements were repeated with an FIO_2_ of 12% (Table S2). Figure 6 shows a representative record of the haemoglobin oxygen saturation obtained on three individual mice in compartments 1, 3 and 5. These results indicate that the profiles were similar for all three mice. The mean (±SD) results of the 7 animals are reported in Table S2. There were no significant differences among mice located in different compartments in terms of latency or time to nadir. Concerning the amplitude of the change in saturation, the results from the first, second and third cycles differed from one another when the hypoxic phase lasted 40 seconds. The values of the three cycles were, therefore, not averaged and comparisons of amplitude of desaturation or resaturation were made for the third cycle. There were no significant differences related to the position in the box.

**Figure 7:**
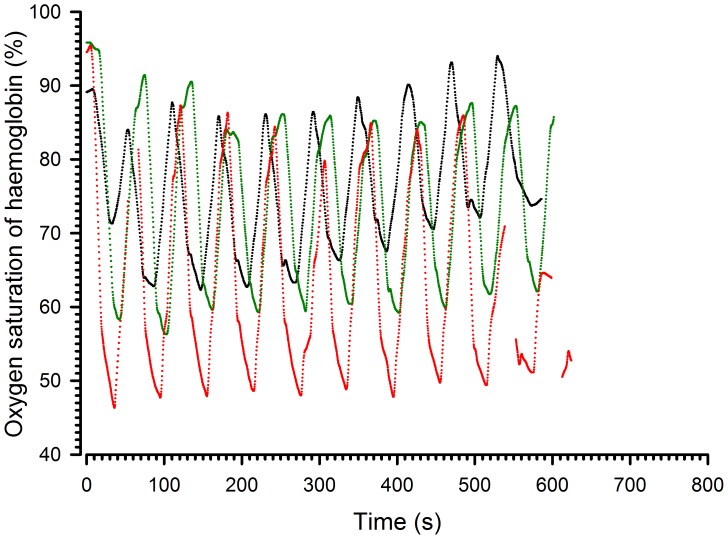
Representative records of haemoglobin oxygen saturation during chronic hypoxia-reoxygenation. Cycles alternated 30 s of normoxia and hypoxia. The black line corresponds to a hypoxic FIO_2_ of 12%, the green line to a FIO2 of 9 % and the red line to an FIO_2_ of 6%.

Finally, do all these parameters persist during a long period of cyclic hypoxia? In order to determine the effect on saturation of a prolonged cyclic exposure, animals were exposed to 30 second hypoxia – 30 second normoxia cycles (at FIO_2_ 6%, 9% and 12%) and to 15 second hypoxia – 45 second normoxia cycles (at same FIO_2_) for 10 minutes. Figure 7 shows a representative record of the haemoglobin oxygen saturation during cycles alternating 30 second of normoxia and hypoxia. As indicated previously, the amplitude of desaturation was dependent on the FIO_2_. When looking at the time effect, the amplitude was the same throughout the 10 cycles at 12% FIO_2_ and decreased significantly from the first to the second and from the first to the fourth cycle at 9% and 6% FIO_2_, respectively. Thereafter, the amplitude of desaturation remained stable. The decreases in amplitude between the first and subsequent cycles were, however, limited and reached 4 and 5%, respectively.

**Figure 8:**
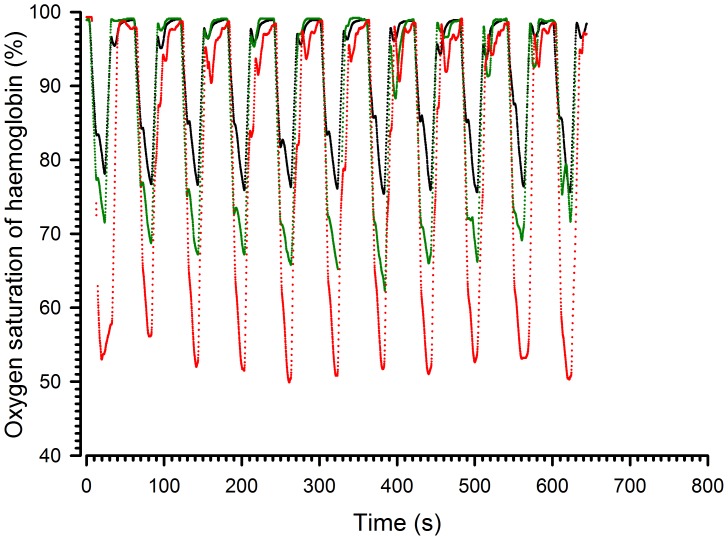
Representative records of haemoglobin oxygen saturation during chronic hypoxia-reoxygenation. Cycles alternated 15 s of hypoxia – 45 s of normoxia. The black line corresponds to a hypoxic FIO_2_ of 12%, the green line to a FIO_2_ of 9% and the red line to an FIO_2_ of 6%.

Regarding cycles alternating 15 second of hypoxia – 45 second normoxia (Figure 8), the results indicated that the amplitude of the 10 cycles remained stable at 12%, 9% and 6% FIO2.

## Discussion

The aim of the present work was to provide proof of concept of a device enabling mice to be subjected to standardised, reproducible and fully controlled cycles of intermittent hypoxia. For this purpose, several requirements had to be met:

### a. Homogeneity of the Conditions in the Different Compartments

CFD simulations helped us to design the size and geometry of the splitter plates at the inlet of the chamber in order to supply the 5 compartments homogenously. Computed simulations were satisfactory, but it was not possible to introduce all potential parameters (e.g., the presence of a moving animal) into this theoretical model. A biological validation was, therefore, performed. This suggested that the design of the device was adequate because the pattern of desaturation-resaturation was similar in the different compartments, in terms of the timing and the amplitude of desaturation. Animal movements, which may influence the flow, had a negligible effect.

### b. Set up of Input Parameters to Achieve Hypoxia and Reoxygenation

The open loop strategy proved to be a good choice because it is easily programmable (easy changes in required parameters, such as oxygen level, cycle length or number) and enables good control of the hypoxia-reoxygenation cycles. There are, nevertheless, some limitations related to mechanical and electronical constraints. Firstly, the analogue flowmeter valve measures flows between 0–10 l/min both for air and nitrogen, thus enabling, at most, a combined flow of 20 l/min to be measured. Secondly, the nitrogen compressor provides nitrogen with a pollution level less than 0.5% only for a maximum flow of 10 l/min. Thirdly, minimum times required for DC valve opening limit the minimum length of the control period and, therefore, the minimum time required to obtain the desired gas concentration. Hence, 3 seconds were needed to achieve concentrations of 20% oxygen starting from a minimal level for the first loop and 4 seconds for the second loop. On the other hand, 18 seconds were needed to downregulate the oxygen level from maximum to minimum levels for the first loop and 11 seconds for the second one. It would be possible to improve these times, but this would submit mice to acoustic stresses that could interfere with the experiments. Nevertheless, these value are sufficient to generate a cyclic variation of oxygen pressure with a rhythm as high as 100 per minute and an FIO_2_ as low as 6%.

### c. *In vivo* Consequences

When a hypoxic gas mixture was breathed in, the haemoglobin oxygen saturation decreased first rapidly, then more progressively. The nadir observed was proportional to the FIO_2_ (Figure 5) and was as low as 50% when the FIO_2_ was only 6%. The nadir was not dependent on the duration of the hypoxic phase (the shortest duration tested was 15 sec). In contrast, the amplitude of resaturation was drastically influenced by the length of the normoxic phase. When the normoxic phase was 45 seconds (see Table S1), the amplitude of resaturation was identical to the amplitude of desaturation meaning that the animal returned to normal saturation values at the end of the first cycle of hypoxia-reoxygenation. When reduced to 30 or 20 seconds, the duration of the normoxic phase was not long enough to allow complete resaturation and the amplitude of desaturation and resaturation then differed. During the subsequent cycle, the amplitude of desaturation thus decreased even if the nadir of saturation was slightly lower. The swing of oxygen pressure in the body is, therefore, reduced when the reoxygenation time is too short. This effect was present but limited when the time was 30 seconds or more. Although a rhythm of cyclic hypoxia above 100 per minute is technically feasible, profound hypoxia–reoxygenation is difficult to generate with a frequency greater than 90 per minute.

In the obstructive sleep apnoea syndrome, it has now been well demonstrated that the frequency of apnoea is directly associated with the consequences of the disease (cardiovascular morbidity and mortality). This disease is generally considered as severe when the apnoea-hypopnoea index (the number of desaturation-resaturations) is more than 30 per hour of sleep and this index can reach a value close to 100 [2]. The amplitude of desaturation is also important. It has been shown that the systemic inflammation associated with sleep-disordered breathing is proportional to the nadir of haemoglobin oxygen saturation and nadir values less than 70% are observed [9]. These extreme clinical situations are similar, in terms of cycles of hypoxia-reoxygenation and severity of desaturation, to the ranges achievable with the present device.

In the literature, describing chronic intermittent hypoxia rat models where a fall of the arterial oxyhaemoglobin saturation was induced to levels of ≈ 60 % to 80 % when animals were exposed to FIO2 of 3–5% during 15 seconds [10], [11]. Thus, amplitudes of desaturation obtained with our device are comparable with the reported systems used to induce hypoxia – reoxygenation cycles in rats given that the arterial saturation of our mouse model falls to levels of ≈ 50% to 80% in function of FIO2 (12%, 9% or 6%).

In conclusion, we have described a device that enables mice to be submitted to chronic intermittent hypoxic phases with all animals submitted to the same conditions. We also set up conditions allowing effective phases of oxygen depletion and supplementation to be generated as measured by arterial oxygen saturation levels. Thanks to its unique technical approach using laminar flows and infusion of pre-determined gas mixtures, this system can very precisely generate various environments imitating pathologies of different severities. This device has several important perspectives for the future. Indeed, using a murine model, we will be able to mimic sleep apnoea, which can be the result of several factors, affects millions of people, and is independently associated with increased cardiovascular risk. This model will allow us to monitor and analyse phenomena that are known or suspected to be associated with the condition, including production of reactive oxygen species, subsequent oxidative stress, inflammation, and skeletal muscle dysfunction, and also help in emerging studies, for example to investigate the role of hypoxia in the regulation of the immune system.

## Supporting Information

Table S1 **Evolution of haemoglobin oxygen saturation as a function of the FIO_2_ and duration of the hypoxic phase.**
(PDF)

Table S2 **Effect of position on the change in haemoglobin oxygen saturation.**
(PDF)
