# Supplementary material for: A New Device to Mimic Intermittent Hypoxia in Mice
Source: PLoS One. 2013 Apr 2;8(4):e59973. doi: 10.1371/journal.pone.0059973 (PMC3615002; doi:10.1371/journal.pone.0059973)
Supplement: Table S1 — Evolution of haemoglobin oxygen saturation as a function of the FIO2 and duration of the hypoxic phase. (PDF) [file pone.0059973.s001.pdf]

Table S1: Evolution of haemoglobin oxygen saturation as a function of the FIO_2 and duration of the hypoxic phase.

		**Cycle 1**		**Cycle 2**		**Cycle 3**		**Cycle 4**	
		15 s hypoxia	45 s normoxia	20 s hypoxia	40 s normoxia	30 s hypoxia	30 s normoxia	40 s hypoxia	20 s normoxia
**Latency (s)**	FIO_2 12 %	3.61 ± 0.6	10.4 ± 0.9	3.99 ± 0.5	10.3 ± 0.8	4.58 ± 0.7	10.8 ± 1	4.81 ± 0.7	8.75 ± 1.4
	FIO_2 9 %	3.2 ± 0.7	8.9 ± 0.9	4.7 ± 0.7	8.8 ± 0.9	2.9 ± 1	8.5 ± 0.9	4.6 ± 0.8	6.2 ± 1.7
	FIO_2 6 %	2.3 ± 0.4	11.2 ± 0.7	2.7 ± 1.3	10.7 ± 1	2.9 ± 2	9.5 ± 0.9	5.9 ± 0.6	7.4 ± 1.5
**Time to peak (s)**	FIO_2 12 %	21.78 ± 1*	38.6 ± 0.8 *	26.3 ± 1 *	33.63 ± 1.4 *	36.2 ± 0.9*	24 ± 1.1 *	43.9 ± 0.8*	11.2 ± 1.4*
	FIO_2 9 %	20.7 ± 1*	40.8 ± 0.7 *	24.1 ± 1 *	34.1 ± 1.3 *	35.7 ± 1.1*	26.1 ± 0.9*	41.6 ± 1.7*	13.8 ± 1.7 *
	FIO_2 6 %	23.9 ± 0.9*	36.5 ± 1.3 *	28 ± 1.3 *	32.2 ± 1.8 *	36.6 ± 2.3*	27.8 ± 1*	40.1 ± 2*	12.6 ± 1.5*
**Amplitude of change (%)**	FIO_2 12 %	-19.9 ± 1.7 $^{\$}$	20.7 ± 1.6 $^{\$}$	-23.3 ± 2.7 $^{\$}$	22.9 ± 2.5 $^{\$}$	-24.8 ± 1.6 $^{\$}$	22.1 ± 2 $^{\$}$	-24.5 ± 2$^{\$}$	11.25 ± 1.52†$^{\$}$
	FIO_2 9 %	-28 ± 1.9 $^{\$}$	28.4 ± 2.1 $^{\$}$	-30.9 ± 2.3 $^{\$}$	30 ± 1.9 $^{\$}$	-32.6 ± 2.1 $^{\$}$	28.1 ± 2.2 $^{\$}$	-29.6 ± 2.2$^{\$}$	17 ± 2.8†$^{\$}$
	FIO_2 6 %	-41.5 ± 2.1 $^{\$}$	40.1 ± 2.2 $^{\$}$	-40.1 ± 1.9 $^{\$}$	36.7 ± 2.1 $^{\$}$	-39.4 ± 1.8 $^{\$}$	35.9 ± 2 $^{\$}$	-33.8 ± 3.3 $^{\$}$	16.1 ± 2.4†$^{\$}$

Saturation was measured during four consecutive cycles of 60 s during which the hypoxic phase was progressively lengthened (15, 20, 30 and 40 s). Latency, time to peak change and amplitude of change were measured in 7 animals (mean±se) at different FIO_2 (6, 9 and 12%). Comparisons were made using a one-way ANOVA followed by Newman-Keuls test. p <0,05 * between each hypoxic phase duration, † between 40 s hypoxic phase and the others, $^{\$}$ between each FIO_2.
