# Supplementary material for: A New Device to Mimic Intermittent Hypoxia in Mice
Source: PLoS One. 2013 Apr 2;8(4):e59973. doi: 10.1371/journal.pone.0059973 (PMC3615002; doi:10.1371/journal.pone.0059973)
Supplement: Table S2 — Effect of position on the change in haemoglobin oxygen saturation. (PDF) [file pone.0059973.s002.pdf]

Table S2: Effect of position on the change in haemoglobin oxygen saturation.

		15s hypoxia / 45s normoxia		20s hypoxia / 40s normoxia		30s hypoxia / 30s normoxia		40s hypoxia / 20s normoxia	
	Position	Desaturation	Resaturation	Desaturation	Resaturation	Desaturation	Resaturation	Desaturation	Resaturation
**Latency (s)**	Box 1	3.8 ± 0.7	11.4 ± 0.5	3.7 ± 0.7	10.8 ± 0.7	5.4 ± 1	10.8 ± 0.7	8.4 ± 1.3	10.6 ± 0.4
	Box 3	3.4 ± 0.6	11.5 ± 1	4.3 ± 0.4	12.2 ± 1.4	4.4 ± 0.4	11.7 ± 1	9.5 ± 1.5	7.7 ± 2.2
	Box 5	1.8 ± 1.2	12.4 ± 0.6	4.1 ± 1.2	11.7 ± 0.7	5.9 ± 0.8	10.2 ± 1.5	11 ± 0.7	7.7 ± 1.8
**Time to nadir (s)**	Box 1	22.6 ± 0.6	42.3 ± 3.8	27.2 ± 0.4	33.5 ± 0.4	35.3 ± 0.7	24.8 ± 0.8	42.2 ± 1.2	10.8 ± 1.4
	Box 3	23.1 ± 0.9	36.8 ± 0.8	27.9 ± 1.2	31.4 ± 1	37.2 ± 0.8	24.4 ± 0.8	39 ± 2.5	13.1 ± 2.1
	Box 5	24.7 ± 0.5	35.5 ± 0.6	27.6 ± 1.2	32.9 ± 0.3	34.3 ± 1.8	26.7 ± 1.6	36.7 ± 2.3	16.7 ± 2
**Amplitude of change (%)**	Box 1	-19.6 ± 1.1	19.2 ± 1.6	-22.3 ± 0.6	22 ± 0.6	-23.8 ± 1.6	22.6 ± 1.1	-16.9 ± 1.4*	9.5 ± 2*
	Box 3	-27.1 ± 2	26.7 ± 2	-25.7 ± 2	25.6 ± 2.3	-22.5 ± 1.3	21.1 ± 1.6	-14.9 ± 3*	12.3 ± 5. 2*
	Box 5	-26 ± 1.3	25.4 ± 1.9	-25.2 ± 2	25.6 ± 2	-23.4 ± 1	24.3 ± 1.5	-13.6 ± 0.7*	11.7 ± 2*

Values of latency, time to peak change and amplitude of change measured during the third cycle with 4 different hypoxic-phase durations. FIO_2: 12%. Values are expressed as mean± SD of 7 animals. One-way ANOVA followed by Newman-Keuls test was used for comparison. There were no statistically significant differences according to position; * indicates significant difference ($p<0.05$) vs the other hypoxic phase durations.
